# Research on evaluation of Wuhan air pollution emission level based on entropy weight method

**DOI:** 10.1038/s41598-024-55554-z

**Published:** 2024-02-29

**Authors:** Na Wang, Yaxin Zhang

**Affiliations:** HuBei University, Wuhan, China

**Keywords:** Entropy weight method, Wuhan, Air pollution, Emission level assessment, Climate sciences, Environmental sciences

## Abstract

For the lack of precise monitoring and accurate assessment models for air quality, this paper fully considers such constraints and establishes an evaluation model of air pollution emission level to evaluate the air pollution emission level of Wuhan—a city in central China. The model uses entropy weight method including important indicators of air pollution into the integrated optimization of air quality assessment, laying the basis for sources of pollution and the reasonable and effective city development. The total emissions of air pollution for Wuhan shows a gradual upward trend over time, mainly coming from industrial pollution. The government can reduce air pollution by focusing on detecting major polluting industries, promoting industrial technological progress and innovation, and strengthening the effective implementation of emission trading system.

## Introduction

With China's rapid economic growth and urbanization development, air pollution has become a serious problem and has received continuous public attention. Air pollution, such as PM_2.5_ and sulfur dioxide (SO_2_) have significant adverse effects on environment. Shapiro^1^ shows impressively, no matter in the period of imperialism or Mao Zedong, China has not avoided the change of natural environment and the fact that human oppression and violence against nature exist at the same time. Industrialization and urbanization aroused by the increase of population in county, the improvement of consumptive level, the acceleration of mineral and ore mining, increasing long-distance transportation and expanding air pollution have led to the increase of air pollution. The continuous growing momentum of car ownership, the increase in proportion of durable goods have also increased the pressure on the environment. The innovation and development of technology and management, the entry into the global market, the improvement of national environmental protection organisations have also promoted the improvement of resource utilization efficiency. Many efforts have been given to find the new methods and models for evaluation and measurement, such as Zhao and Yuan^2^.

China's environmental protection originated in the late 1970s, with similar characteristics and some problems with other centrally planned economies for all environmental protection system. These problems include restrictions on citizens' right to participate, lack of independent environmental protection campaigns or non-governmental organizations, too few responses to international agreements, organizations and institutions, high attention to the central state power, restrictions on the behavior of private organizations, excessive attention to technological development, and coordination between national authorities and departments, and restrictions on the authority of the environmental organisations^3–5^. For the environmental protection, the air pollution control began after 1972. With the continuous promotion of environmental protection institutions and legislations, China's air pollution monitoring system is becoming more and more completed. Since 2010, smog has occurred frequently in many places in China, and air pollution has become the focus of the government and society. Amidst a transformative economic milieu in China, domestic enterprises are venturing into the global market, exposing them to intensified perils in international trade and investment^6^. Meanwhile, the industrial sector has always been regarded as the industry with largest energy consumption, and air pollution is derived mainly from industrial exhaust^7^.

Taking this economic and social background as basis, and for the lack of precise monitoring and accurate assessment models for air quality, this paper takes the air pollution emission that means the emission of exotic substances which enter the near-surface or lower atmosphere in gaseous form as the research theme. The air pollution emission include nitrogen oxide, sulphur and carbon oxides, as well as floating dust and suspended particles, sometimes including formaldehyde, radon and various organic solvents, which have adverse effects on the human body or the ecosystem. Using the entropy weight method to evaluate the air pollution emission level of air quality for Wuhan in China.

Below is the arrangements of the latter part. Firstly, we review the documents and raise the research quesions. After giving a review of literatures on air pollution, it puts forward the key scientific problems of this paper. The third part explains the sample source and data. Based on the detailed description of the sample selection, it explains the key indicators selected and the reasons for selection in detail. The fourth part constructs the entropy method model, introducing the relevant theories of entropy method and constructs the model. The fifth part is data standardization preprocessing and entropy calculation, calculating the entropy weight of air pollution in Wuhan. The sixth part, puts forward countermeasures and suggestions for energy consumption in Wuhan providing strong theoretical support and practical guidance for the formulation of air pollution emission reduction system. And the last two parts are the conclusions and discussions.

## Literature review and research questions

Since the late 1960s, western developed countries began to pay considerable attention on the relationship between economic growth and environment pollution. Mishan^8^ first put forward the defect of economic growth, which clearly shows the adverse impact which economic growth gives to environment. Hirsch^9^ and Schumacher^10^ expressed the same concern. The United Nations World Environment Conference held in Stockholm in 1972 is one of the results of this growing concern. At this meeting, people found that development is more important than environment in their goal ranking. Slowing economic growth in order to protect the environment seems to be not a priority on the developing countries’ schedule, thereby reducing so-called poverty related pollution^11^.

Ari^12^ paid attention to the impact of air pollution on buildings, and analyzed the cost of pollution. His research shows that the sum of decoration cost and living cost loss composing the whole pollution, and there is a “comprehensive doles kauff function” relationship between the repair cost of pollution damage and pollution. The important variables in air pollution are particle income and concentration, and the correlation with sulfur dioxide (SO_2_) is not very clear. Randy^13^ showed that the rearrangers of “standard” air pollutants subject to more strict control (because the county level does not meet the national ambient air quality standard) usually have higher expenditure on reducing air pollution (APA). On average affected factories increase operating costs by hundreds of thousands of dollars a year to reduce certain pollutants.

The follow-up research literature focuses on air pollution, air pollution assessment, climate change, policy on energy security assessment and its impact on the economy. Johannes et al.^14^ proved that the combination of air pollution, climate change, policy on energy security and their combined strength can bring many positive effects. Tamir and Joseph^15^ found that pollution caused by air has a negative impact on stock returns, and it may even influences local traders. Nichol et al.^16^ showed that the biggest industrial factor causing external costs is coal-fired power generation, with a loss rate of 0.8–5.6 times added value. Litao et al.^17^ showed that the main pollutant in the cities of China is PM_10_. They believe the API system should include more pollutants to fully reflect the air quality of Chinese cities and guide future air pollution control. Yansui et al.^18^ pointed out that if population density to be higher, then the use of energy will be lower around the China, but its impact on environment depends on the types of pollutants. Jiabei et al.^19^ pointed out that Wuhan's air quality has improved in small amounts in recent years, but pollution levels remain high. Chuangwang et al.^20^ found that increased efficiency can reduce air pollution in general. Mian et al.^21^ suggested that metropolis transport investment increases SO_2_ emissions shortly, as it may lead to road congestion and increase emissions from low-speed transport. However, seeing from a long way, investment in urban transport makes a positive influence on reducing sulphur dioxide emissions, as it can widen roads and let transport systems to be more convenient. In addition, they found a Simon Kuznets curve for sulfur dioxide in China, where environmental controls are effective in curbing sulfur dioxide emissions. In 2019, the European Commission unveiled the European Green Deal^22^, a new plan that seeks to eliminate greenhouse gas emissions by 2050 and supporting a resource-independent economy^23^.

In recent years, there are also scholars concerned on the influence for Covid-19 on air pollutant emissions. Silva et al.^24^ pointed that air pollutions is a global concern, accelerating the degradation of medieval historic buildings and having a detrimental effect on human health. Progiou et al.^25^ showed that significant reductions in emissions from road, sea and air transport, ranging from 20 to 90 per cent. Papadogiannaki et al.^26^ found that in the first case, the carbon dioxide emissions from these projects have increased by more than 40 percent compared to Columbia, while in the second case, measures such as telecommuting, virtual participation and digitizing bureaucratic processes have reduced emissions by at least 20 percent. The study shows that these measures can significantly reduce greenhouse gas emissions after a COVID-19 pandemic. Riaz and Farid^27^ explained that Improving the effectiveness of green supply chains is a critical step towards minimizing waste, optimizing resource use, and reducing the environmental impact of business operations.

The focus on pollution emission caused by air and its impact on economic growth began in 2010 by Chinese scholars. Zhou et al.^28^ found that the change direction of three kinds of air pollutants is the same with the overall output value of enterprises, and the larger the overall output value is, the larger the amount of air pollutants is. Based on the concept of ecological civilization, Guan^29^ made a comprehensive evaluation on the development level of urban ecological civilization by using the entropy method, the evaluation index system is proved to be reasonable and feasible in practice. Wang et al.^30^ studied the pollution distribution characteristics of various regions in China. The research showed that the relationship between degree of regional pollution emission caused by air and concentration of polluting industries is strong. We must pay more attention to coordinated growth of regional industrial structure and comprehensive environmental treatment, and finally achieve the target of reducing the pollution level by decreasing the emission of polluting industries. Feng and Yao^31^ showed that air pollution is positively affected by economic level, scale effect, technology effect and energy consumption structure. Niu and Cui^32^ showed that the network infrastructure’s construction can restrain the pollution caused by air, promoting the upgrading of urban industrial structure and improve the city's technological innovation ability.

From above we can find that how to correctly evaluate the degree of air pollution, for putting forward appropriate economic development strategies and formulating appropriate social development policies has become the key element to ensure the sustainable development. In this paper, we take air pollution emission to be research theme and take air pollution emission of Wuhan in recent 20 years as the research sample, using the method of entropy to evaluate the emission level of air pollution comprehensively, in order to put forward reasonable countermeasures and suggestions.

## Sample source and data description

**Figure 1:**
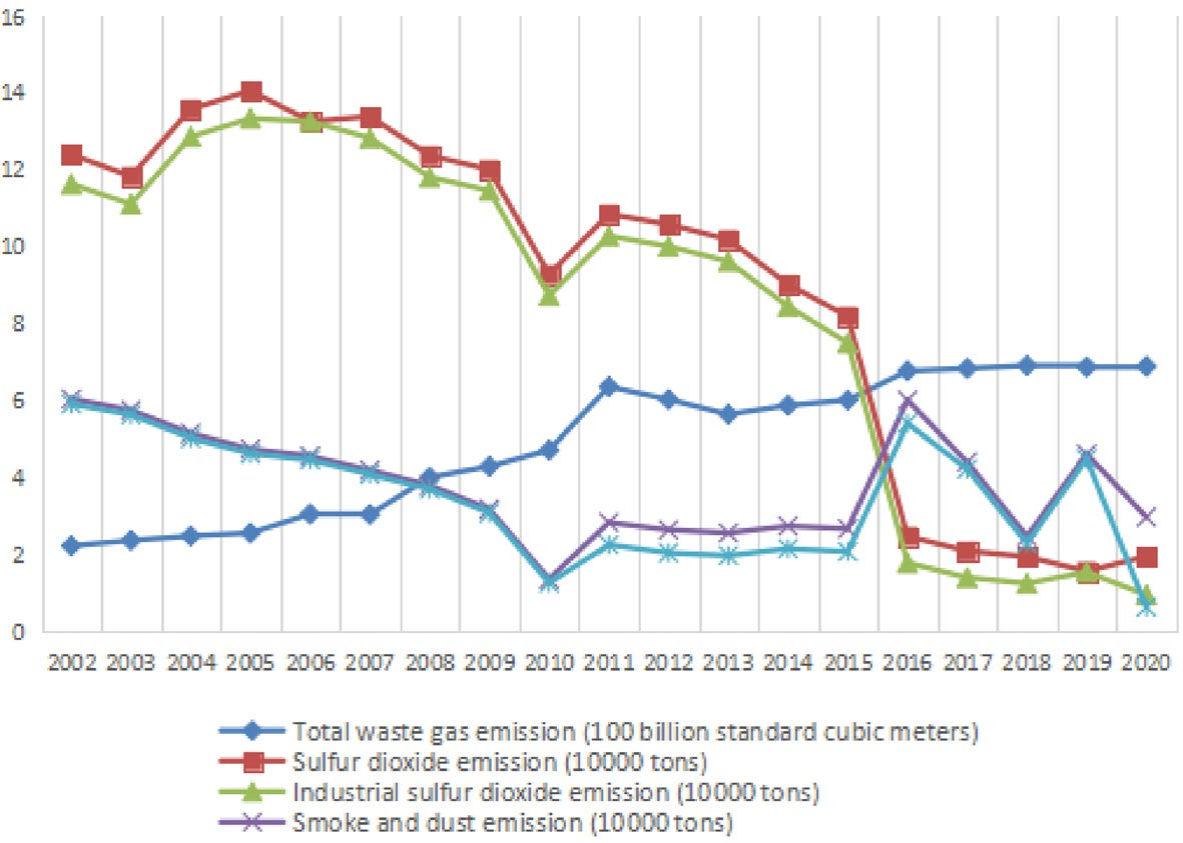
Statistics of air pollutant emission in Wuhan.

As a central megacity with the strategy of rising in Central China, Wuhan is typical and representative for China. For this reason, we select the air pollution emission data of Wuhan from 2002 to 2020^33^ as the research sample. Among the existing statistical data in China, the statistics of air pollution emission data mainly focus on the level of industrial air pollutants’ emission. For the analysis of air pollution’s emission level in Wuhan and considering the availability of data, the total amount of smoke and dust emission, waste gas emission, SO_2_ emission in Wuhan are selected. A total of 95 original data were finally obtained. See Table 1 and Fig. 1 for detailed original data.

**Table 1:** emission data of air pollutants in Wuhan.

Y	Total waste gas emission (100 billion standard cubic m)	SO_2_ emission (10,000 t)	Industrial SO_2_ emission (10,000 t)	Smoke and dust emission (10,000 t)	Industrial smoke and dust emission (10,000 t)
2002	2.23491	12.4	11.63	6.04	5.91
2003	2.37493	11.83	11.11	5.75	5.63
2004	2.48942	13.58	12.86	5.15	5.03
2005	2.56736	14.06	13.34	4.75	4.63
2006	3.06035	13.26	13.26	4.57	4.47
2007	3.04992	13.39	12.83	4.2	4.09
2008	4.0147	12.37	11.81	3.81	3.71
2009	4.29987	12.01	11.46	3.19	3.09
2010	4.7208	9.28	8.73	1.37	1.27
2011	6.35995	10.85	10.28	2.84	2.26
2012	6.02781	10.58	10.01	2.65	2.05
2013	5.64177	10.19	9.62	2.57	1.98
2014	5.8732	9.02	8.45	2.75	2.16
2015	6.01105	8.19	7.5	2.68	2.08
2016	6.77049	2.48	1.79	6.01	5.41
2017	6.83542	2.1	1.41	4.41	4.23
2018	6.9094	1.95	1.26	2.48	2.3
2019	6.87241	1.57	1.55	4.62	4.49
2020	6.890905	1.96	0.96	2.98	0.64

In order to more clearly analyze the changes of air pollutant emissions in Wuhan from 2002 to 2020, the trend chart of air pollutant emissions in Wuhan is drawn according to the original data given in Table 1 (see Fig. 1 for details). From Fig. 1, we can find that SO_2_ emission fluctuates greatly and has an obvious downward trend, especially in 2016, with a significant decline of about 57,000 t, and then the decline tended to be flat; The emission of smoke and dust show a downward trend as a whole. From 2002 to 2015, the overall change trend is relatively slow, but the change range is large from 2016 to 2020. It increased sharply by about 33,000 t in 2016, decreased sharply by about 33,000 t from 2017 to 2018, increased by about 20,000 t in 2019, and decreased sharply in 2020; On the whole, the total emission of waste gas in Wuhan showed a slow upward trend from 2002 to 2020. There was a sudden increase in 2011, with an increase of about 164 billion standard cubic meters, with a large growth range. After 2016, the total amount of waste gas emission increased slowly, the total emission of air pollutants basically tended to be flat; The emission change trend of industrial SO_2_ and industrial smoke and dust in air pollutants closely coincides with the emission change trend of SO_2、_smoke and dust respectively, indicating that these three air pollutions are mainly coming from industry; We can see a remarkable decreasing in air pollutant emissions in 2020, coinciding with the research results showed as Progiou et al.^25^ and Papadogiannaki et al.^26^ suggested.

## Entropy approach model construction

There are many methods to evaluate air pollution, and many researchers have gotten rich conclusions. Brodny and Tutak^34^ used Kohonin's artificial neural network model to similarly group EU countries according to the specific gases and dust they emit into the atmosphere, using a sample of EU countries. Progiou^35^ identified the most suitable measurement method and tacticses for Thessaloniki and Greece to tackle particulate air pollution, including the calculation of industry emissions and the application of air quality simulation systems. Khan and Wang^36^ offers a multi-criteria decision-making (MCDM) methodology to successfully showcase the suggested technique.

We aim to identify the key components of Wuhan's air pollutant emissions, and to provide relevant advice on environmental management. And just as Chen^37^ said, entropy approach is a weighting method with objectivity. The weight of the index is determined from calculating the index’s entropy. To avert the error aroused by the influence of human factors in the evaluation. So we used entropy approach as our research method.

### Entropy approach Introduction

Entropy can be used to explain the event information’s average amount. Mathematically, entropy can be seen as the expected amount of information contained in an event. Entropy used to judge degree of discretization of the index. After mastering and collecting the data in origin, weight is determined accordingly. Entropy can impact index information comprehensively.

### Model construction

#### Collection and sorting of original data

Assuming that x is the matrix of Wuhan atmospheric environment assessment system corresponding to m assessment years and N assessment indicators, there are:

1 $${\text{X}} = \left[ {\begin{array}{*{20}c} {\begin{array}{*{20}c} {\begin{array}{*{20}c} {x11} & {x12} \\ \end{array} } \\ {\begin{array}{*{20}c} {x21} & {x22} \\ \end{array} } \\ \end{array} } & \cdots & {\begin{array}{*{20}c} {x1n} \\ {x2n} \\ \end{array} } \\ \ldots & \ldots & \ldots \\ {\begin{array}{*{20}c} {xm1} & {xm2} \\ \end{array} } & \cdots & {xmn} \\ \end{array} } \right],$$

where X = {*xij*}*m* × *n*, (0≤*i*≤*m*,0≤j≤n), where i is the selected year, j is the unselected evaluation index, and Xij stands for the value of the j evaluation index in the i-th year.

#### Normalization processing

Before constructing the comprehensive index according to the equal weight, it is must to implement standardized process and assimilation processing on the data index. The standardized processing is used to eliminate the units between each index and the number and size of the same index. The assimilation processing is used to change the nature of the inverse index so that all indicators have the same force direction on the evaluation system. According to the properties of the index, the range method is used to normalize the indexes. The formula for normalizing each index is as follows:

2 $${x}_{ij}^{\mathrm{^{\prime}}}=\frac{{x}_{ij}-{\text{min}}({x}_{ij})}{{\text{max}}({x}_{ij}-{\text{min}}({x}_{ij})},$$

a standardized matrix can be obtained:

3 $${\text{X}}^{\prime } = \left[ {\begin{array}{*{20}c} {\begin{array}{*{20}c} {\begin{array}{*{20}c} {x_{11}^{\prime } } \\ {x_{21}{\prime} } \\ \end{array} } & {\begin{array}{*{20}c} {x_{12}^{\prime } } \\ {x_{22}^{\prime } } \\ \end{array} } \\ \end{array} } & \cdots & {\begin{array}{*{20}c} {x_{1n}^{\prime } } \\ {x_{2n}^{\prime } } \\ \end{array} } \\ \cdots & \cdots & \cdots \\ {\begin{array}{*{20}c} {x_{m1}^{\prime } } & {x_{m2}^{\prime } } \\ \end{array} } & \cdots & {x_{mn}^{\prime } } \\ \end{array} } \right],$$

where ${x}_{ij}{\prime}$ is the transformation value of the j-th index in the i-th year; i = 1, 2,…, m, m is the number of evaluation years; j = 1,2,…,n, n is the number of evaluation indicators.

#### Index weight’s determination

Suppose ${r}_{ij}$ is the proportion of air environmental pollutant emissions in Wuhan in each year in the past 19 years, then:

4 $${r}_{ij}=\frac{{x}_{ij}^{\mathrm{^{\prime}}}}{\sum_{i=1}^{m}{x}_{ij}^{\mathrm{^{\prime}}}}.$$

#### Determination of index entropy

Suppose ${{\text{e}}}_{j}$ is the determination of index entropy in Wuhan in each year in the past 19 years, then:

5 $${e}_{j}=-\frac{1}{lnm}\sum_{{\text{i}}=1}^{{\text{m}}}{{\text{r}}}_{{\text{ij}}}{\text{ln}}({{\text{r}}}_{{\text{ij}}}).$$

#### Determination of index weight

Suppose ${{\text{w}}}_{j}$ is the determination of index weight in Wuhan in each year in the past 19 years, then:

6 $${w}_{j}=\frac{1-{e}_{j}}{\sum_{j=1}^{n}(1-{e}_{j})}.$$

#### Index evaluation value of each year

Suppose ${{\text{p}}}_{i}$ is the index evalution value in Wuhan in each year in the past 19 years, then:

7 $$p_{i} = \mathop \sum \limits_{j = 1}^{n} r_{ij} w_{j} .$$

**Figure 2:**
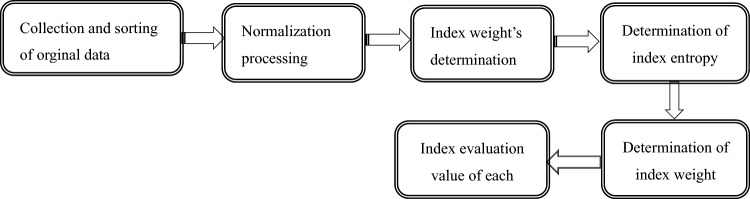
the method arrangement.

The method arrangement can be seen in Fig. 2:

## Data standardization preprocessing and entropy calculation

Through the standardized preprocessing of the data in Table 1, the annual pollution emission level is measured, we can see it in Table 2.

**Table 2:** Air pollution emission data after preprocessing.

Y	SO_2_ emission (10,000 t)	Industrial SO_2_ emission (10,000 t)	Smoke and dust emission (10,000 t)	Industrial smoke and dust emission (10,000 t)
2002	0.0000	0.8619	1.0000	1.0000
2003	0.8215	0.8199	0.9379	0.9469
2004	0.9616	0.9612	0.8094	0.8330
2005	1.0000	1.0000	0.7238	0.7571
2006	0.9359	0.9935	0.6852	0.7268
2007	0.9464	0.9588	0.6060	0.6546
2008	0.8647	0.8764	0.5225	0.5825
2009	0.8359	0.8481	0.3897	0.4649
2010	0.6173	0.6276	0.0000	0.1195
2011	0.7430	0.7528	0.3148	0.3074
2012	0.7214	0.7310	0.2741	0.2676
2013	0.6902	0.6995	0.2570	0.2543
2014	0.5965	0.6050	0.2955	0.2884
2015	0.5300	0.5283	0.2805	0.2732
2016	0.0729	0.0670	0.9936	0.9051
2017	0.0424	0.0363	0.6510	0.6812
2018	0.0304	0.0242	0.2377	0.3150
2019	0.0000	0.0477	0.6959	0.7306
2020	0.0312	0.0000	0.3448	0.0000

Based on the standardized pretreatment of the original data, the weight of each index comprehensive evaluation is calculated, and the results are shown in Table 3.

**Table 3:** Weight of air pollution emission indicators.

Pollution index	SO_2_ emission (10,000 t)	Industrial SO_2_ emission (10,000 t)	Smoke and dust emission (10,000 t)	Industrial smoke and dust emission (10,000 t)
weight	0.3539	0.2804	0.1751	0.1907

From Table 3 we can find among air pollution emission indicators’ weights in Wuhan, the weight of SO_2_ emission indicators ranks first, with a weight of 0.3539, followed by industrial SO_2_ emission indicators with a weight of 0.1907, next are industrial smoke and dust emission indicators with a weight of 0.1907 and smoke and dust emission indicators with a weight of 0.1751; It showed that SO_2_ and industrial SO_2_ have a great influence on the level of air pollution, followed by smoke dust and industrial smoke dust in Wuhan.

**Figure 3:**
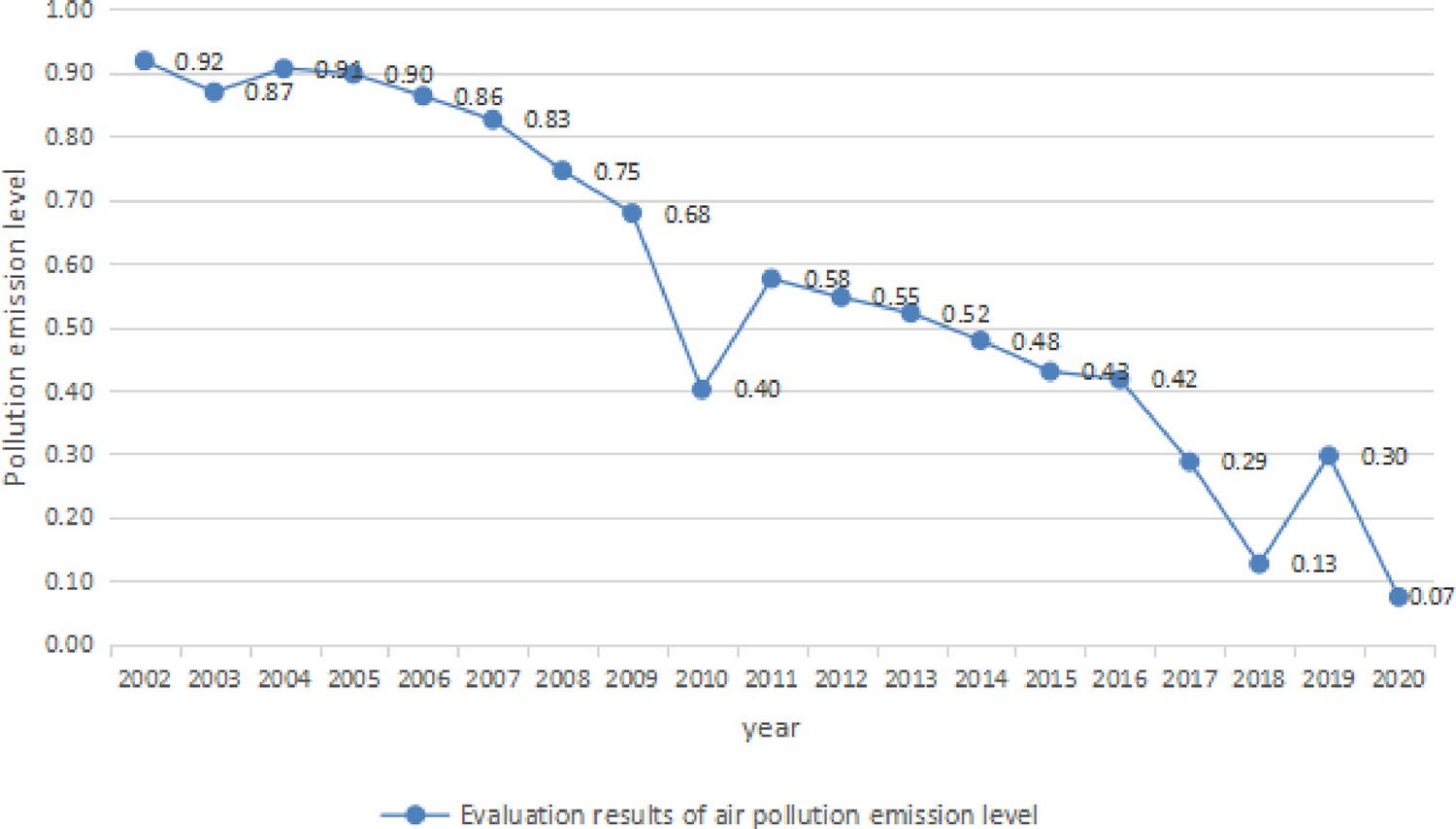
evaluation results of air pollution emission level in Wuhan.

Through the weight and preprocessing data, the annual air pollution emission level of Wuhan can be obtained, as shown in Fig. 3.

From Fig. 3 we can find the air pollution level in Wuhan showed a downward trend as a whole, decreased to 0.4 in 2010 increasing to 0.58 in 2011 and decreased slowly from 2011 to 2016. It showed a significant fluctuation from 2016 to 2020. On the whole, the evaluation results of air pollution emission level in Wuhan decreased from 0.92 in 2002 to 0.07 in 2020, indicating that the trend of sustainable development of atmospheric environment in Wuhan is good.

**Figure 4:**
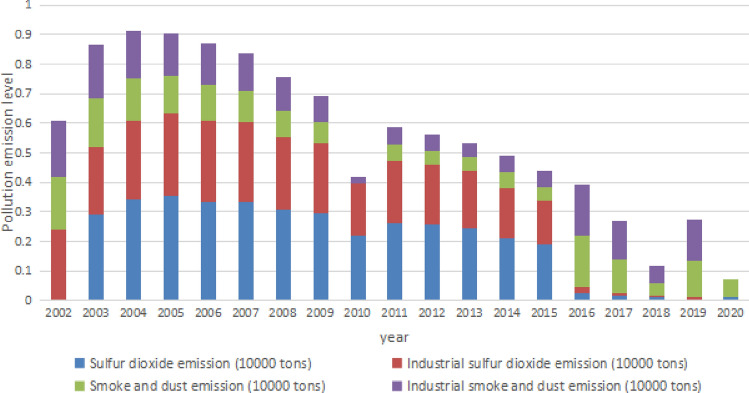
annual contribution of each index to air pollution emission level.

Through the preprocessing data in Table 2 and the weight data in Table 3, the annual contribution of index to the air pollution emission level are shown in Fig. 4.

From Fig. 4 we can see that from 2002 to 2004, the air pollution level in Wuhan showed a slight upward trend, and began to decline continuously. By 2020, the air pollution level has dropped to a lower level. Industrial waste gas emission still has a important influence on the emmision level of air pollution. It is particularly important to focus on reducing industrial waste gas emission while controlling waste gas emission as a whole.

## Conclusions

Based on the relevant data of the basic situation of atmospheric environmental protection in Wuhan from 2002 to 2020, we make an empirical study on the air pollution emission level in Wuhan. The results are as follows:

First, the overall number of pollution gas emission in Wuhan shows a gradual upward trend over time. After reaching the highest level in recent years, the overall number of pollution gas emission basically does not continue to rise, but the overall number of pollution gas emission is at a high level yet, which indicates that Wuhan's economic growth has also brought about the increasing emission of air pollution;

Second, the total smoke, dust emission, SO_2_ emission, and SO_2_ emission from industry and smoke from industry and dust emission in Wuhan generally show a downward trend. Among them, the total SO_2_ emission and total dust and smoke emission fluctuate greatly. After the sudden decline of total SO_2_ emission in 2016, the emission basically tends to be stable and the emission level is low. The overall emission of dust and smoke increased sharply in 2016 and fluctuated greatly in the following years, indicating that concern has been put to air pollution in process along with economic development;

Third, the most important phenomenon is that total emission of smoke, SO_2_ and dust, industrial SO_2_ emission and smoke from industry and dust’s emission account for a large proportion of the total emission respectively, indicating that the air pollution in Wuhan mainly comes from industrial pollution;

Fourth, among the weights of air pollution emission indicators in Wuhan, the weight of SO_2_ emission indicators and industrial SO_2_ emission indicators are relatively large, indicating that the weight of these two indicators has the largest dispersion in all four indicators, contains the most evaluation information, and has a great impact (weight) on the comprehensive evaluation of air pollution in Wuhan;

Fifth, the overall pollutant emission level in Wuhan shows a downward trend, indicating that while paying attention to economic growth, we should pay more attention to emission reduction and reducing fossil energy consumption. From the comprehensive evaluation results and various pollutant emission indicators, it can be found that the total pollutant emission is gradually decreasing and the environmental pollution level is constantly improving.

Wuhan is one of six provinces in central China, and the air pollution’s level in China as a whole can be seen from the city. Our objetive is to find the balance between environmental protection and economic development, so as to better guide the practice of economic green development in China. Of course, many factors affect air pollution’s emission level, including energy Communities in mitigating air pollution^38^, stakeholder analysis and perceptions^39^, and participation in co-designing policies and measures^40^. These factors may affect the air pollution emission and the environmental protection’s effects. Lack of analysis of other majoe factors on the influence of air emissions is the limitation in this study, it is also the direction of future research.

We need learn more methods to monitor and evaluate the air pollution, including integrating fuzzy k-clustering and fuzzy neural network^41^, blockchain^42^, artificial intelligence^43^, Neutrosophic Linguistic valued Hypersoft^44^, DIBR II-BM-CoCoSo MCDM Model^45^, Fuzzy Soft Matrices-Based Algorithm^46^.

## Discussions

Based on the above findings and analysis results, in order to reduce air pollution and environmental pressure, we puts forward the following policy suggestions:

First, focus on monitoring major polluting industries. We can focus on the main industries with high polluting air emissions. Sulphur dioxide emissions should be monitored with a focus on the supply of chemical raw materials, electricity, heat, non-metallic minerals and products, ferrous smelting and rolling, textiles and chemical fibres. The monitoring of flue gas emissions should focus on the production and supply of electricity, heat, non-metallic mineral products, chemical raw materials and chemical products manufacturing, textiles, Ferrous smelting and calendering, chemical fibre manufacturing, etc.. For dust emissions, it is necessary to focus on monitoring the non-metallic mineral products sector and the Ferrous and rolling industries. We should regulate and control through industrial and environmental policies, guide the rational allocation of resources, vigorously develop high-tech industries and service industries, and reduce environmental pollution^28^.

Second, promote industrial technological progress and innovation. By accelerating ultra-low emission transformation, strengthen the end treatment of dust removal, denitration and flue gas desulfurization. Through the implementation of measures such as flue gas circulation of sintering machine head and clean transportation transformation, the increased pollution gas emission of terminal treatment is offset, making coordinated treatment between pollution reduction and reduction^47^.

Third, strengthen the effective implementation of emission trading system. The pilot strategy of trading for SO_2_ emission in 2007 not only has a significant emission reduction effect, but also has a long-term sustainable effect^48^. From the perspective of energy consumption, emission trading system can effectively decrease energy use per unit and increase energy consumption^49^. In addition, the pilot strategy of trading for SO_2_ emission in 2007 not only produced an environmental dividend of 11%, but also did not damage the economic interests of enterprises. At the same time, the policy effect of emission trading system is more obvious in areas with higher degree of marketization^50^.

The improvement of energy consumption and utilization efficiency is the key area to achieve emission reduction and green economic transformation and development. We should formulate policies and measures as a whole, promote the improvement of environmental quality from focusing on end treatment, paying more attention to source prevention, achieving synergy, and achieving a win–win situation of air quality improvement and economic development. These are the directions and implications that need attention in the future of this study.

## Data Availability

The DATASETS used and/or analysed during The current study available from The corresponding author on reasonable request.
